# Nonlinear Ion‐Specific Control of Underwater Adhesion by Bottlebrush Polymer Interfaces

**DOI:** 10.1002/advs.78071

**Published:** 2026-09-28

**Authors:** Chang‐Sheng Wang, Hui Guo, Hu Zhang, Duy Anh Pham, Nahid Hassanpour, Wojciech Raj, Qiang Peng, Justine Saber, Tzu‐Hsuan Huang, Florina G. Halmac, Xavier Banquy

**Affiliations:** ^1^ Faculty of Pharmacy Université De Montréal Montréal Québec Canada; ^2^ Biomedical Engineering Institute Faculty of Medicine Université De Montréal Montréal Québec Canada; ^3^ Department of Chemistry Faculty of Art and Science Université De Montréal Montréal Québec Canada

**Keywords:** bottlebrush polymers, hydration‐mediated interactions, ion‐specific effects, underwater adhesion

## Abstract

Programming interactions at aqueous interfaces remains challenging because structured hydration layers resist molecular contact and obscure the role of dissolved ions. As a result, electrolytes are often treated as passive screening agents rather than active design parameters. Here, we show that electrolyte identity can deterministically regulate the mechanical state of soft interfaces through ion‐specific hydration effects. Using quaternized bottlebrush polymers on mica as a force‐resolved model system, we identify a two‐channel mechanism that separates polymer‐substrate interactions from interpolymer cohesion. Cation hydration controls access to surface binding sites: strongly hydrated Na^+^ preserves polymer anchoring by remaining solvated near the surface, whereas weakly hydrated K^+^ directly occupies lattice sites and suppresses adhesion. In parallel, anion valency activates a distinct cohesion pathway, where multivalent anions bridge polymer chains across the confined interface. Coupling between these pathways produces non‐additive behavior, enabling regimes in which substrate anchoring is suppressed while interpolymer bridging sustains adhesion. These findings establish electrolyte composition, not simply ionic strength, as a primary control parameter for interfacial mechanics, providing a framework for designing adaptive adhesion and hydration‐mediated interactions in aqueous environments.

## Graphical abstract

The efficiency of underwater adhesion is modulated by ion‐specific effects. Strongly hydrated Na^+^ remains separated from the mica surface by its hydration shell, preserving access to binding sites, whereas weakly hydrated K^+^ can partially dehydrate and occupy ditrigonal cavities. This ion‐specific site occupation suppresses polymer adsorption and displaces chains away from the surface, promoting extended conformations capable of interfacial bridging.

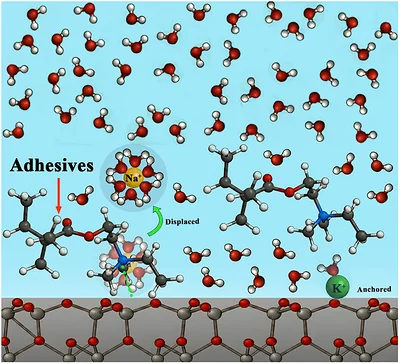

## 1 Introduction

Underwater adhesion is central to technologies ranging from marine coatings and biomedical sealants to soft robotics and colloidal assembly [1, 2, 3, 4]. Its significance is further amplified by rapidly emerging applications at bioelectronic interfaces, where achieving long‐term, stable attachment of implantable sensors, bioelectrodes, and related devices in hydrated physiological environments remains a fundamental challenge [5, 6, 7]. Several strategies have been developed to address this challenge, including chemical functionalization with mussel‐inspired adhesive groups such as DOPA [8], molecular and structural designs inspired by mussel foot proteins [9, 10], and the in situ formation of permanent covalent bonds to establish stable coupling between bioelectronic devices and biological tissues [11, 12].

Although these molecular and structural strategies can achieve strong and durable adhesion in aqueous environments, the underlying interfacial mechanisms remain incompletely understood. Electrostatic interactions are attenuated by ionic screening, while structured hydration layers at interfaces resist molecular contact [2, 3, 4]. Dissolved ions further complicate this landscape by simultaneously modulating screening and reorganizing interfacial hydration structures [13, 14, 15]. Nevertheless, ions are often treated as a passive background in adhesive design, with salt concentration adjusted primarily to tune screening strength, while ion identity and hydration thermodynamics remain largely overlooked. Elucidating these coupled effects is therefore essential not only for understanding molecular interactions at hydrated interfaces but also for establishing mechanistic design principles for next‐generation underwater adhesive materials.

Ion‐specific hydration determines how closely ions and surfaces can approach one another and thereby modulates the resulting nanoscale surface forces that govern adhesion. Cations and anions differ not only in charge and size, but also in hydration strength (free energy and structure), which determines whether they interact with interfaces through hydration‐retaining outer‐sphere associations or partially dehydrated, more contact‐like configurations with surface functional groups [15, 16, 17, 18, 19, 20]. Weakly hydrated ions can more readily reorganize interfacial water and access surface sites, whereas strongly hydrated ions tend to stabilize hydrogen‐bonded water networks that reinforce hydration barriers [21, 22, 23, 24, 25, 26]. However, predicting these effects in realistic adhesion systems remains difficult because multiple ion‐dependent processes occur simultaneously, including surface binding, electrostatic screening, and polymer conformational responses. As a result, the distinct roles of cations and anions, and the conditions under which they interact synergistically to regulate adhesion, remain poorly resolved, despite their importance in systems such as ion binding on minerals [27, 28], cation‐π interactions [2, 29, 30, 31] and hydration‐lubricated contacts [32, 33, 34, 35].

To address this challenge, we establish a force‐resolved model system based on bottlebrush polymers (BBPs) adsorbed on mica to disentangle ion‐specific pathways governing polymer–substrate binding and interpolymer interactions. A dilute electrolyte regime (ionic strength ≤ 10 mm) was selected to preserve measurable electrostatic interactions and enable the respective contributions of direct polymer‐mica attraction and ion‐mediated interpolymer bridging to be experimentally distinguished before strong double‐layer compression obscures their force signatures. Under confinement, small ion‐induced perturbations are directly amplified into measurable changes in interfacial mechanics that reflect redistribution of polymer segments between surface‐bound trains and mid‐gap extending loops and tails [36]. This sensitivity is particularly advantageous on lattice‐defined mineral substrates such as mica, where binding requires access to discrete surface sites whose availability is controlled by cation hydration, providing a direct handle on hydration‐gated substrate accessibility. In addition, BBP architecture further enables control of interfacial structure through backbone length and surface coverage. At low coverage, shorter backbones tend to adopt more flattened adsorption conformations [37, 38, 39], reducing structural complexity and facilitating the isolation of ion‐specific effects.

To implement this approach, we study quaternized BBPs (q‐BBPs) bearing permanently charged ammonium side chains using a surface forces apparatus (SFA). By comparing asymmetric (q‐BBP/mica) and symmetric (q‐BBP/q‐BBP) configurations across two backbone lengths, we decouple polymer‐substrate interactions from interpolymer effects. Within this framework, we identify two coupled pathways, which we call channels, governing adhesion. Channel 1 describes polymer‐substrate interactions, jointly regulated by polymer‐side charge screening and substrate‐side accessibility controlled by cation hydration. Channel 2 captures interpolymer cohesion mediated by ion bridging. Because the mica lattice also carries a permanent structural charge, both pathways remain effectively pH‐independent within our experimental window (pH ≈ 6–11) yet can be independently tuned or strongly coupled. Furthermore, bulk ion association regulates the availability of bridging anions, giving rise to non‐additive behavior that cannot be explained by ionic strength alone. Together, this framework establishes electrolyte composition as an active control parameter governing interfacial interactions and the mechanics of underwater adhesion.

## 2 Results and Discussion

### 2.1 Precision Synthesis and Structural Characterization of Cationic Bottlebrush Polymers with Tunable Architectures

The q‐BBPs were synthesized following protocols established in previous work [40, 41]. In brief, the two q‐BBPs were prepared via a “grafting‐from” approach (Figure 1a). Poly(methyl methacrylate‐*co*‐2‐(2‐bromoisobutyryloxy)ethyl methacrylate) (P(MMA‐*co*‐BiBEM)) backbones were synthesized via atom transfer radical polymerization (ATRP) of methyl methacrylate (MMA) and trimethylsilyl‐protected 2‐hydroxyethyl methacrylate (HEMA‐TMS). By adjusting the monomer‐to‐initiator (bromide) ratio and quenching the polymerization reaction at low conversion (≈20%) to minimize dispersity, we obtained backbones with degrees of polymerization (DP_bb_) of ≈ 418 and ≈ 880 (see details in Table S1).

**FIGURE 1:**
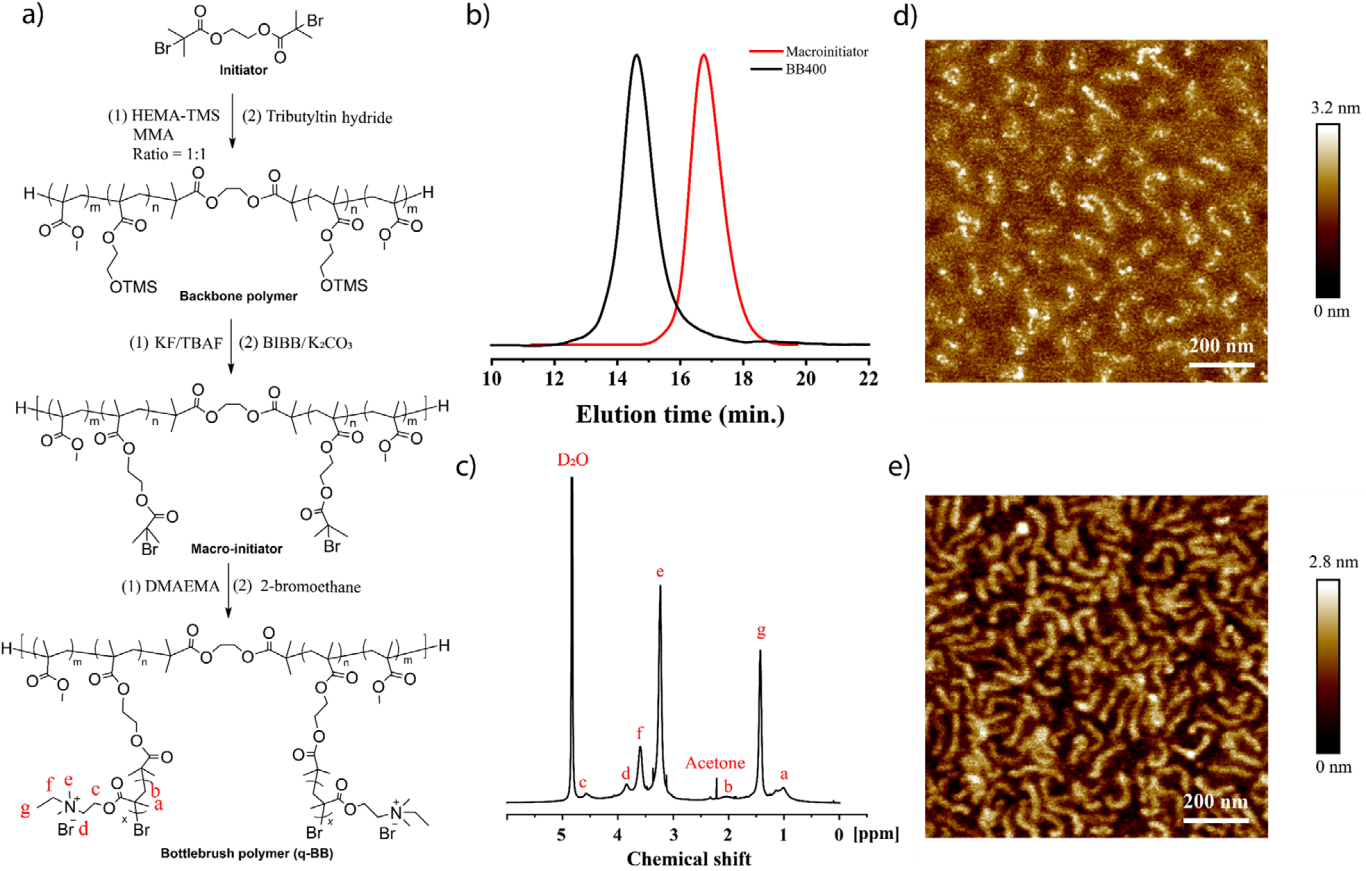
Synthesis and molecular characterization of q‐BBPs. a, Synthetic route for q‐BBPs via atom transfer radical polymerization (ATRP). P(MMA‐co‐BiBEM) backbones were prepared from a methyl methacrylate/trimethylsilyl‐protected 2‐hydroxyethyl methacrylate (MMA/HEMA‐TMS) feed, targeting degrees of polymerization of ≈ 400 and ≈ 800. PDMAEMA side chains were grown from the macro‐initiators via grafting‐from ATRP and subsequently quaternized to yield cationic brushes. b, Gel permeation chromatography (GPC) traces of the P(MMA‐*co*‐BiBEM) macroinitiator (black) and the resulting BB400 (red). The shift to shorter elution times confirms successful side‐chain grafting and increased molar mass. c, ^1^H NMR spectrum of q‐BBP in D_2_O, confirming side‐chain incorporation, and quantitative quaternization of the ammonium groups. d,e, Representative atomic force microscopy (AFM) height images (PeakForce Tapping in air) of isolated q‐BBP molecules adsorbed on mica for d, BB400 and e, BB800. Worm‐like morphologies were observed, with contour lengths of *L*_c_ = 70.2 ± 19.3 nm for BB400 and *L*_c_ = 150.6 ± 25.4 nm for BB800, scaling with the backbone degree of polymerization. Scale bars: 200 nm.

The trimethylsilyl protecting groups were subsequently removed with tetrabutylammonium fluoride (TBAF) to expose pendant hydroxyl groups, which were esterified with 2‐bromo‐2‐methylpropanoyl bromide to introduce ATRP initiators along the backbone, yielding P(MMA‐*co*‐BiBEM) macroinitiators. Poly(2‑(dimethylamino)ethyl methacrylate) (PDMAEMA) side chains were then grown from these macroinitiators, producing side‐chain degrees of polymerization of DP_sc_ ≈ 76 for BB400 and DP_sc_ ≈ 60 for BB800. A final one‐step quaternization yielded the cationic BBPs P(MMA_220_‐*co*‐BiBEM_198_)‐*g*‐qPDMAEMA_76_ (BB400) and P(MMA_462_‐*co*‐BiBEM_418_)‐*g*‐qPDMAEMA_60_ (BB800).

Gel permeation chromatography (GPC) traces of the P(MMA_220_‐*co*‐BiBEM_198_) and P(MMA_462_‐*co*‐BiBEM_418_) copolymers revealed a clear shift toward higher molecular weight following side‐chain grafting. The resulting BBPs exhibited single, unimodal, and approximately symmetric peaks, without resolved secondary peaks or shoulders at longer elution times, while maintaining relatively low dispersities (*M*_w_/*M*_n_ = 1.28) (Figure 1b and Table S2). ^1^H NMR spectroscopy further supported successful side‐chain incorporation and quantitative quaternization (Figure 1c). To minimize contributions from weakly and reversibly adsorbed low‐molecular‐weight species, the polymer‐coated mica surfaces were rinsed 4–5 times with ultrapure water before the SFA measurements. The measured interfacial responses are therefore expected to arise predominantly from the adsorbed BBPs.

Atomic force microscopy (AFM) imaging (Figure 1d,e) revealed distinct, uniform and worm‐like morphologies, with contour lengths of *L*_c_ = 70.2 ± 19.3 nm for BB400 and *L*_c_ = 150.6 ± 25.4 nm for BB800. The contour‐length ratio of these contour lengths (≈ 2.1) closely matches the backbone DP ratio, supporting the structural fidelity of the synthesis. Although the side‐chain DPs of BB400 and BB800 differ by approximately 21%, this difference is considerably smaller than the approximately twofold differences in their backbone DPs and AFM‐measured contour lengths. Backbone length therefore represents the more pronounced architectural difference between the two polymers. Accordingly, the differences observed between BB400 and BB800 are interpreted as being primarily associated with, rather than exclusively caused by, backbone length. Nevertheless, because side‐chain length was not independently controlled, its contribution to the measured interfacial behavior cannot be fully excluded.

### 2.2 Baseline Interactions in the Absence of Added Electrolytes

Before introducing specific electrolytes, it is essential to establish the intrinsic mechanical response of the q‐BBP system in pure water. We therefore characterized both asymmetric (q‐BBP/mica) and symmetric (q‐BBP/q‐BBP) configurations to define a comprehensive “zero‐salt” reference state. In the asymmetric configuration, the interaction isolates polymer‐substrate adhesion, establishing the baseline affinity of q‐BBP for the mica lattice. In contrast, the symmetric configuration isolates polymer‐polymer interactions, allowing us to determine whether opposing layers exhibit a net repulsive or adhesive interaction in the absence of electrolyte. Establishing these baselines is critical for the subsequent analysis, as it provides a reference against which electrolyte‐induced changes in substrate anchoring and interlayer cohesion can be quantitatively evaluated.

#### 2.2.1 Asymmetric Polymer–Mica Interactions

Approach force‐distance profiles in the asymmetric q‐BBP/mica geometry exhibit a pronounced long‐range attraction for both BB400 and BB800 (Figure 2a,b), arising from the electrostatic interactions between the positively charged q‐BBP layer and the negatively charged mica lattice. The attractive onset distance ($D_{\mathrm{onset}}^{\mathrm{att}}$) increases with q‐BBP concentration and is consistently larger for BB800, reflecting its more extended overall architecture (Figure 2c). At the highest coverage (*C_p_* =  200 µg mL^−1^), $D_{\mathrm{onset}}^{\mathrm{att}}$ reaches ≈ 82.5 ± 8.3 nm for BB400 and ≈ 107.5 ± 10.6 nm for BB800. This trend indicates a coverage‐controlled extension of the interaction range, with molecular architecture further modulating its spatial reach.

**FIGURE 2:**
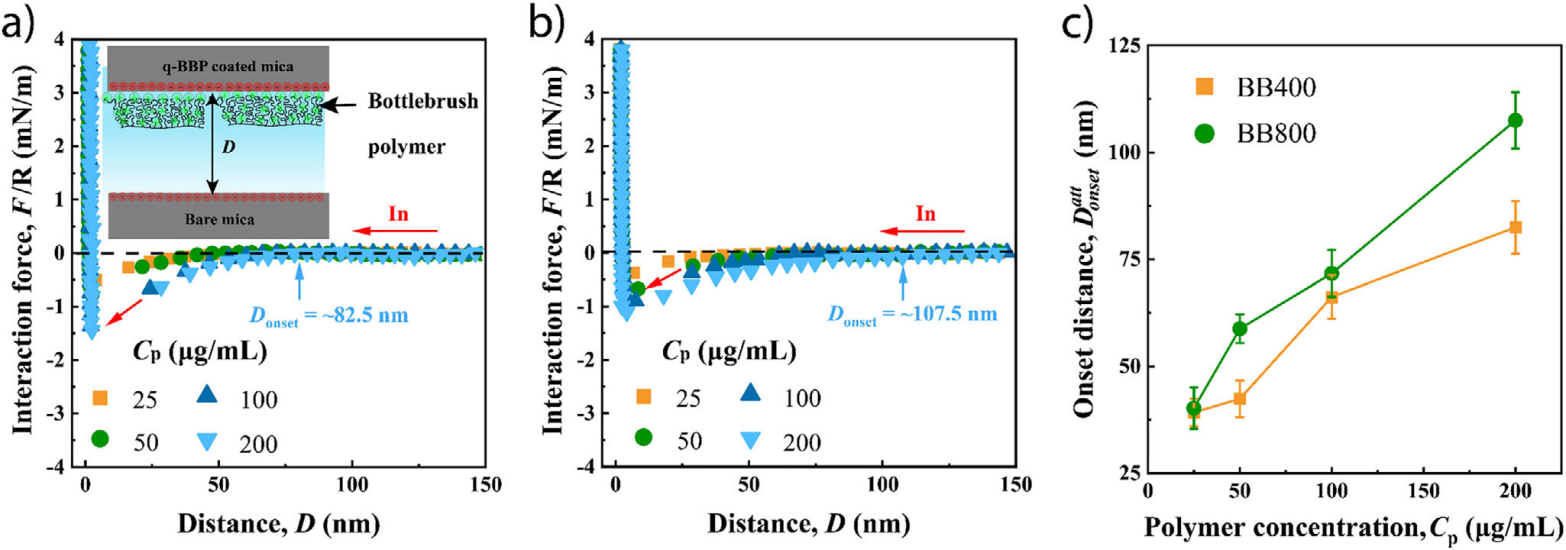
Asymmetric (q‐BBP/mica) interaction forces in pure water. a, b, Representative SFA normal force‐distance profiles, *F*(*D*)/*R*, measured between a q‐BBP coated mica surface and bare mica after polymer incubation at varying concentrations (*C_p_* =  25,  50,  100,  and 200 µg mL^−1^) for a, BB400 and b, BB800. The approach force profiles reveal a long‐range attractive interaction between the two surfaces. Inset in (a) shows a schematic illustration of the asymmetric configuration c, Attractive onset distance ($D_{\mathrm{onset}}^{\mathrm{att}}$) as a function of polymer concentration, indicating a coverage‐ and backbone‐length‐dependent spatial reach of the interaction. All curves were collected at an approach speed of 1 nm s^−1^. Error bars represent standard deviation from *n* ≥ 3 independent experiments.

This systematic increase in interaction range reflects both enhanced surface charge density and a progressive shift in adsorption conformation. As surface sites become occupied, late‐arriving chains attach through fewer segments, favoring partially adsorbed conformations with loops and tails extending into solution. This behavior follows established train‐loop‐tail descriptions of polymer adsorption, where the bound fraction decreases with increasing surface coverage [42, 43]. In addition, lateral crowding during adsorption promotes out‐of‐plane extension, as steric confinement and entropic penalties disfavor fully flattened configurations [44, 45]. These extended segments displace interacting charges away from the surface and can span the aqueous gap to engage the opposing mica, accounting for both the outward shift of $D_{\mathrm{onset}}^{\mathrm{att}}$ and its stronger amplification for the larger BB800 architecture (Figure 2c). This architectural scaling is further supported by independent measurements on a homologous q‐BBP with a longer backbone (DP_n_ ≈ 1600, matched side‐chain chemistry), which exhibits $D_{\mathrm{onset}}^{\mathrm{att}}\approx 120$ nm at identical conditions [36]. Separation traces (Figure S1) provide complementary mechanical evidence: adhesion increases with concentration and is accompanied by increasing stretch lengths prior to detachment, particularly at *C_p_* =  200 µg mL^−1^, consistent with the presence of extended, partially adsorbed chain segments capable of forming bridging interactions.

#### 2.2.2 Symmetric Interpolymer Interactions

In contrast to the asymmetric case, the symmetric configuration involves interactions between two like‐charged, polymer‐coated surfaces. As the opposing q‐BBP layers approach, electrosteric interactions between them establish a repulsive background. Nevertheless, adhesion can arise through inter‐surface cross‐bridging, in which polymer segments from one surface bind to accessible bare mica sites on the opposing surface. Accordingly, a weak attractive interaction is observed at low polymer concentrations (*C_p_* =  25‐50 µg mL^−1^), where sparse surface coverage leaves sufficient bare mica sites available for bridging (Figure 3a,d).

**FIGURE 3:**
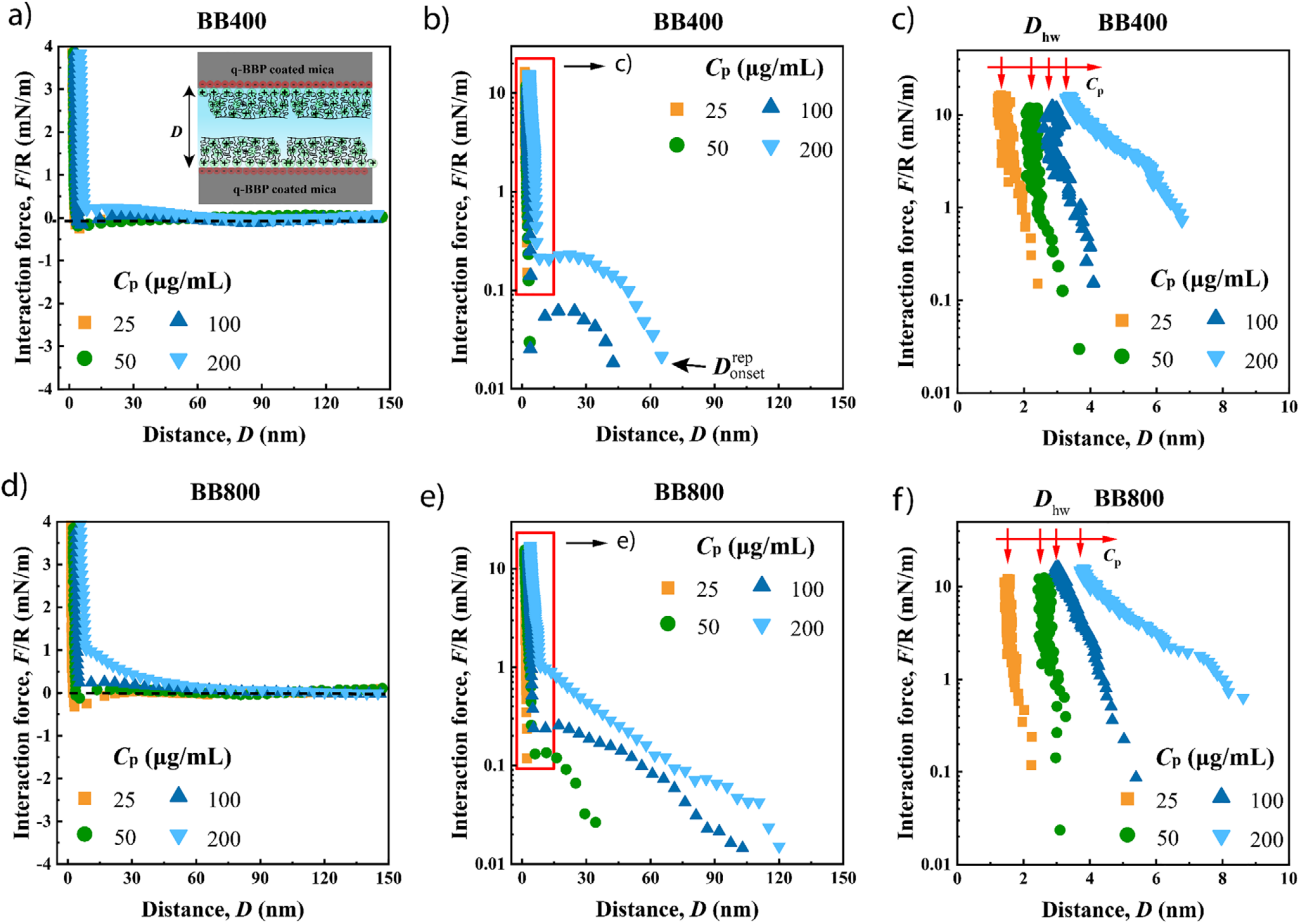
Symmetric (q‐BBP/q‐BBP) interaction forces in pure water. Representative SFA normal force–distance profiles, *F*(*D*)/*R*, measured between two q‐BBP coated mica surfaces at increasing incubation concentrations (*C_p_* =  25,  50,  100,  and 200 µg mL^−1^). a,d, Linear plots for BB400 and BB800 showing a coverage‐dependent transition from weak cross‐bridging‐induced attraction at low *C_p_* to steric repulsion at high *C_p_*. Insert in (a): schematic of the symmetric configuration. b,e, Semi‐log plots highlighting suppression of long‐range attractions and the emergence of far‐field repulsion at high coverage. c,f, Zoomed‐in view of the near‐contact regime. The hard‐wall thickness (*D*_hw_) increases monotonically with *C_p_*, indicating progressively denser adsorbed layers. At the highest concentration (*C_p_* =  200 µg mL^−1^), a distinct repulsive shoulder appears, consistent with steric compression of overlapping brush segments. All curves were collected at an approach speed of 1 nm s^−1^.

As coverage increases (*C_p_* =  100‐200 µg mL^−1^), the availability of bare mica sites progressively diminishes, suppressing cross‐bridging interactions. The interaction correspondingly transitions to a net long‐range repulsion (Figure 3a,b,d,e), and the mechanical response becomes dominated by electrosteric interactions between opposing polymer layers. The onset distance of repulsion ($D_{\mathrm{onset}}^{\mathrm{rep}}$) is consistently larger for BB800 than for BB400 (Figure 3b,e), again reflecting the greater spatial extension inherent to its larger molecular dimensions.

This transition is further evident in the near‐field response (< 10 nm, Figure 3c,f). The hard‐wall thickness (*D*_hw_) increases monotonically with *C_p_*, indicating progressive densification of the adsorbed layers. At the highest concentration (*C_p_* =  200 µg mL^−1^), a distinct repulsive shoulder emerges at separations beyond the hard wall distance observed at lower coverage. This feature is consistent with the onset of steric overlaps between opposing layers, where extended, partially adsorbed bottlebrush segments interact and resist further compression, reflecting the increasing difficulty of interpenetrating densely grafted brushes under confinement.

#### 2.2.3 Decoupling Stable Anchoring from Dynamic Interfacial Coupling

While the force‐distance curves provide structural information on polymer conformation and surface coverage, successive compression‐separation cycles probe the stability and kinetic robustness of these interfacial states. Mechanical reproducibility across cycles serves as a sensitive indicator of whether adhesion is governed by stable polymer‐substrate anchoring or by more dynamic, reconfigurable inter‐surface polymer interactions. By combining adhesion magnitude, hard‐wall thickness (*D*_hw_), and detachment stretch length (*L_s_*), we obtain complementary insight into both the structural organization and the mechanical resilience.

In the asymmetric geometry (q‐BBP/mica), adhesion increases monotonically for both BB400 (from ≈ 30 to ≈ 70 mN m^−1^) and BB800 (from ≈ 50 to ≈ 90 mN m^−1^) at *C_p_* = 25–200 µg mL^−1^ (Figure 4a,d), consistent with increasing surface density of quaternized ammonium groups. Adhesion remains invariant over repeated compression‐separation cycles, and *D*_hw_ shows negligible change (Figure S2), indicating that polymer‐mica interactions are robust and that the adsorbed layers do not undergo significant compaction or reorganization under load. This contrasts with previous observations for much longer bottlebrush polymers (DP = 1600 vs DP = 400 and 800), where steric constraints induced partial desorption and film thinning during cycling [36].

**FIGURE 4:**
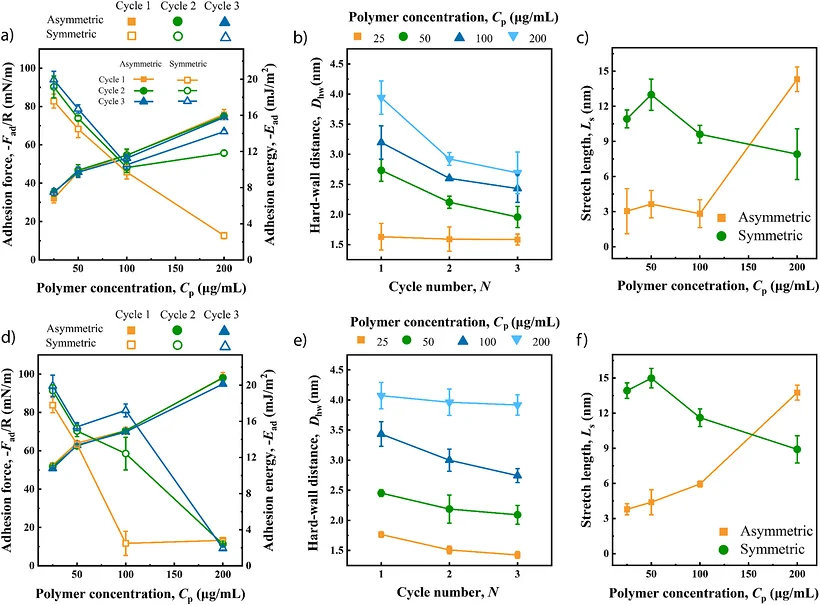
Cycle‐dependent adhesion and chain stretching dynamics. Evolution of adhesion, hard‐wall thickness (*D*_hw_) and detachment stretch length (*L_s_*) measured in pure water over three compression‐separation cycles (approach speed: 1 nm s^−1^). a,d, Adhesion force (‐*F*_ad_/R) and adhesion energy *E*_ad_ as a function of concentration for BB400 (a) and BB800 (d). The asymmetric configuration (solid symbols) exhibits stable, cycle‐independent adhesion, whereas the symmetric configuration (empty symbols) shows adhesion increase after the first compression, indicating force‐induced restructuring. b,e, Hard‐wall thickness *D*_hw_ as a function of cycle number in the symmetric configuration for BB400 (b) and BB800 (e). The concentration‐dependent changes in *D*_hw_ are consistent with different degrees of confinement‐induced rearrangement, whereas the layer formed at 200 µg mL^−1^ undergoes little further compaction. c,f, Stretch length *L_s_* at detachment for BB400 (c) and BB800 (f). Error bars represent standard deviation from *n* ≥ 3 independent experiments.

BB800 consistently exhibits higher adhesion than BB400 across all concentrations. This difference reflects distinct interfacial conformations: BB400 is expected to adopt relatively flattened, train‐dominated structures with a larger fraction of segments directly bound to the surface, whereas BB800 more readily forms partially adsorbed configurations with loops and tails extending into the gap. These extended segments increase the probability of interfacial interactions, leading to enhanced adhesion.

In contrast, the symmetric configuration (q‐BBP/q‐BBP) exhibits a qualitatively different mechanical response. In the first compression cycle, adhesion decreases with increasing *C_p_*, reflecting the progressive reduction of accessible bare mica sites required for inter‐surface cross‐bridging. At *C_p_* = 25–50 µg mL^−1^, adhesion is comparable for BB400 and BB800, indicating that the interaction strength is primarily limited by the site availability rather than the chain architecture. At higher coverage (*C_p_* =  200 µg mL^−1^ for BB400 and =  100 µg mL^−1^ for BB800), adhesion becomes strongly cycle‐dependent, increasing progressively over successive compressions (Figure 4a,d). For example, BB400 adhesion increases from ≈ 15 → ≈ 50 → ≈ 70 mN m^−1^ over 3 cycles, while BB800 shows a similar evolution (≈ 15 → ≈ 60 → ≈ 80 mN m^−1^). This strengthening is accompanied by a systematic decrease in *D*_hw_ (Figure 4b,e), indicating confinement‐driven structural reorganization of the adsorbed layers. We attribute this behavior to progressive conversion of loops and tails into more strongly bound train segments (Scheme S1), enabling closer interfacial contact and enhanced adhesion.

The earlier onset of this transition for BB800 reflects its larger overall structural dimensions, which promotes the formation of outward‐projecting segments that experience confinement at lower surface coverage, facilitating earlier interpenetration and reorganization under compression. In contrast, the flatter BB400 layers require higher coverage to reach a comparable degree of confinement‐induced restructuring. At the highest concentration for BB800 (*C_p_* =  200 µg mL^−1^), *D*_hw_ becomes invariant with cycling, indicating that steric packing within the dense layer limits further structural rearrangement.

Additional insight into the interfacial chain configurations is provided by the detachment stretch length, *L_s_*, which reflects how polymer segments adsorbed on two facing surfaces extend during separation. At low surface coverage (*C_p_* = 25–100 µg mL^−1^), *L_s_* remains short (≈ 3–6 nm, Figure 4c,f) in the asymmetric configuration, indicating predominantly flattened, train‐dominated adsorption (as depicted in Scheme 1a), where detachment is limited by the extension of surface‐bound segments from the side chains. In the symmetric configuration, however, sparse coverage leaves exposed mica sites on both surfaces, enabling inter‐surface cross‐bridging. Detachment then proceeds through progressive peeling of the bottlebrush main chain, producing substantially larger stretch lengths (Figure 4c,f; Scheme 1a). At higher surface coverage (*C_p_* =  200 µg mL^−1^), the behavior diverges. In the asymmetric configuration, *L_s_* increases sharply (up to ≈ 15 nm), reflecting a transition to partially adsorbed conformations with loops and tails extending into the gap (Scheme 1b). These extended segments can bridge the aqueous gap to the opposing bare mica, leading to long‐range peeling during detachment (Figure 4c,f). In contrast, in the symmetric configuration, dense opposing layers generate strong electrosteric repulsion that suppresses interpenetration. As a result, adhesive interactions are restricted to distal chain segments accessing a limited number of residual bare mica sites on the opposing surface. This constrained interaction geometry limits chain extension during detachment, and *L_s_* therefore remains comparatively short despite the increased surface coverage (Figure 4c,f, Scheme 1b). This transition occurs at lower *C_p_* for BB800 than for BB400 (Figure 4c,f), consistent with the greater tendency of longer backbones to adopt extended interfacial conformations.

**SCHEME 1:**
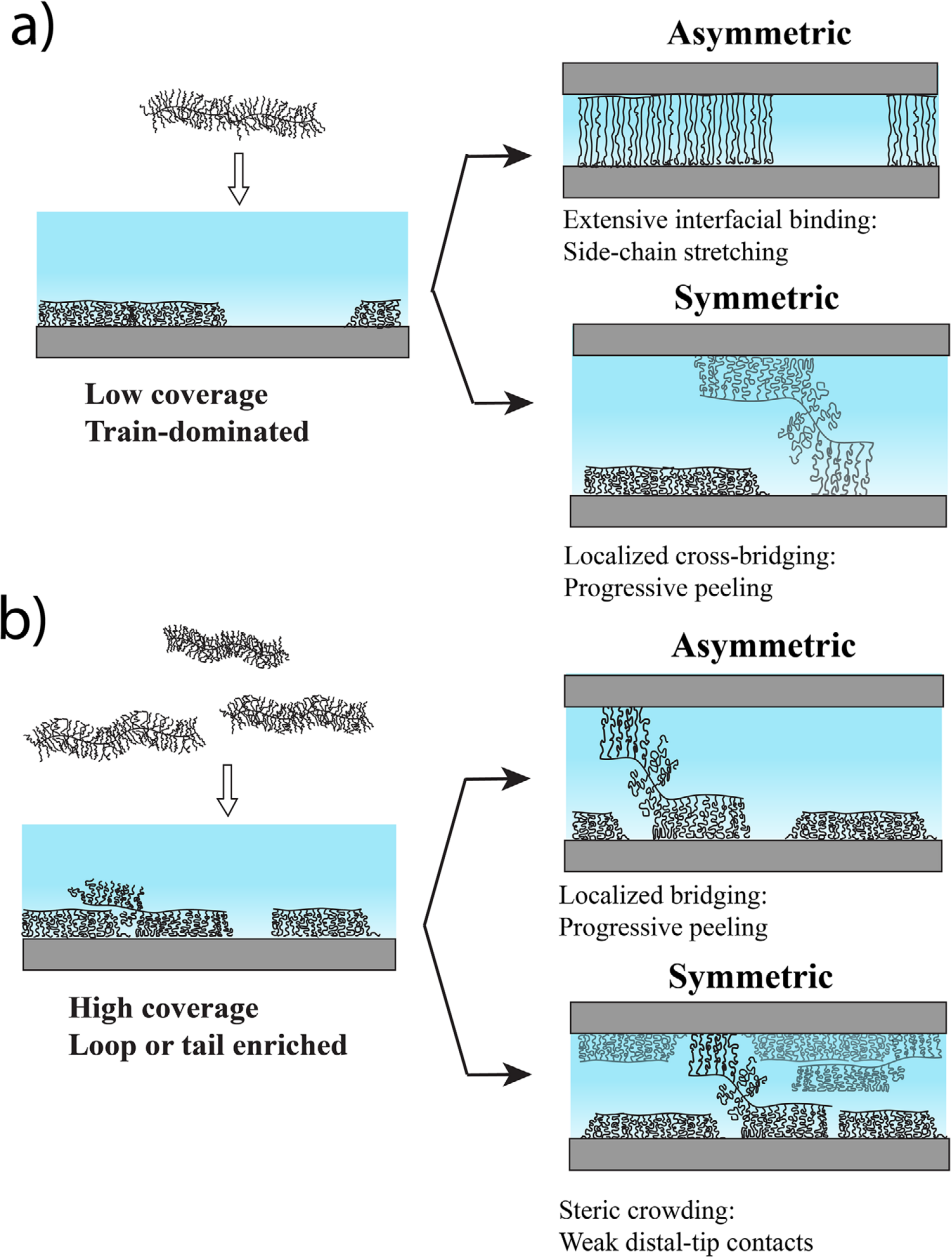
Proposed mechanistic model of surface‐coverage‐dependent q‐BBP conformations and their influence on interfacial detachment dynamics. a, low surface coverage: q‐BBPs predominantly adopt a flattened, train‐dominated conformation on the mica surface. In the asymmetric configuration, the opposing bare mica can establish extensive contact with the adsorbed layer, and detachment is governed by limited extension of surface‐bound segments, resulting in short stretch lengths. In the symmetric configuration, sparse coverage leaves exposed mica regions on both surfaces, allowing polymer chains to form cross‐bridges to localized binding sites on the opposing substrate. Separation proceeds via progressive peeling of these bridged chains, producing significantly longer stretch lengths. b, high surface coverage: Surface crowding promotes partially adsorbed conformations with loops and tails extending away from the substrate. In the asymmetric configuration, these extended segments bridge the aqueous gap to the opposing bare mica, and detachment occurs through progressive peeling of the partially adsorbed chains, leading to substantially larger stretch lengths. In the symmetric configuration, dense bottlebrush layers generate strong electrosteric repulsion that suppresses mutual interpenetration. Adhesion is therefore limited to weak distal‐tip contacts between opposing chains, resulting in comparatively short stretch lengths. Polymer color contrast was employed to distinguish polymers adsorbed on the upper and lower surfaces.

In summary, interfacial adhesion in q‐BBP systems originates from electrostatic attraction between the cationic quaternary ammonium groups and the negatively charged mica surface. At low surface coverage, the interface is dominated by train‐like adsorption, defining a structurally simple regime in which adhesion is governed primarily by polymer‐mica interactions. In the symmetric configuration, apparent bridging arises from polymers connecting opposing surfaces through localized mica binding sites, while direct polymer‐polymer interactions remain repulsive. As surface coverage increases, partially adsorbed loops and tails emerge, introducing steric and electrostatic constraints that progressively limit interfacial contact. This leads to a competition between bridging‐mediated adhesion and electrosteric repulsion. Focusing on the low‐coverage regime therefore establishes a well‐defined baseline in which polymer‐mica interactions dominate, providing a clear reference for isolating ion‐specific effects in subsequent sections.

### 2.3 Ion‐Specific Regulation through Coupled Cation‐Controlled Surface Passivation and Anion‐Mediated Bridging

Having established the mechanical baseline in pure water, we next investigated how the interfacial adhesion is modulated by specific ion identity, namely, cation hydration and anion valency. To account for the different baseline adhesion strengths of BB400 and BB800, the adhesion forces measured in NaNO_3_, Na_2_SO_4_, Na_3_PO_4_, KNO_3_ and K_2_SO_4_ were normalized to the corresponding zero‐added‐electrolyte baseline values reported in Figure 4 (*A*  =  *F*_ad_/*F*_ad,0_). This normalization facilitates comparison of the relative ion‐induced responses of the two polymers. This selection allows independent evaluation of anion valency and hydration (${\mathrm{NO}}_{3}^{-}$ vs ${\mathrm{SO}}_{4}^{2-}$ vs ${\mathrm{PO}}_{4}^{3-}$) as well as cation identity substitution (Na^+^ vs K^+^). For these measurements, q‐BBP concentration was fixed at *C_p_* =  50 µg mL^−1^ for both BB400 and BB800. This condition provides two key experimental advantages: (1) both polymers exhibit comparable baseline adhesion forces in pure water (*F*_*ad*,0_ ≈ 65 mN m^−1^); and (2) the interface rapidly reaches a history‐independent, mechanically equilibrated steady state (Figure 4a,c). These conditions define a stable reference from which ion‐specific modulation of adhesion can be quantitatively resolved.

a, Schematic illustration of the two‐channel adhesion model. Normalized adhesion ($A=\frac{F_{ad}}{F_{ad,\;0}}$) is described as the combined contribution of Channel 1 (polymer–substrate anchoring, modulated by charge screening and cation‐induced passivation) and Channel 2 (interpolymer bridging mediated by multivalent anions). b,c, Influence of anion valency. Normalized adhesion as a function of ionic strength (*I*) for b, BB400 and c, BB800 (*C_p_* =  50 µg mL^−1^) in sodium salts (NaNO_3_, Na_2_SO_4_, Na_3_PO_4_). d, ζ‐potential of BB400 in various electrolytes. The ζ‐potential decreases with increasing anion valency but is insensitive to cation identity (Na^+^ vs K^+^), confirming that K^+^‐induced adhesion loss arises from interfacial passivation rather than bulk polymer collapse. e,f, Cation identity swap (Na^+^ vs K^+^). Adhesion profiles in nitrate and sulfate backgrounds show systematic suppression of adhesion in the presence of K^+^. g, Quantification of the K^+^ penalty ($\Delta_{NO_{3}}^{K}=A_{\mathrm{NaN}O_{3}}\;-A_{\mathrm{KN}O_{3}}$ and $\Delta_{SO_{4}}^{K}=A_{Na_{2}SO_{4}}\;-A_{K_{2}SO_{4}}$), demonstrating a larger reduction in adhesion in nitrate than in sulfate. h, Quantification of the sulfate‐induced bridging gain ($\Delta_{\mathrm{Na}}^{SO_{4}}=A_{Na_{2}SO_{4}}\;-A_{\mathrm{NaN}O_{3}}$ and $\Delta_{K}^{SO_{4}}=A_{K_{2}SO_{4}}\;-A_{\mathrm{KN}O_{3}}$), showing amplified bridging in the K^+^ background. i, Analysis of non‐additivity. The additive prediction (open symbols, Equation 5) consistently underpredicts measured adhesion in K_2_SO_4_, indicating that suppression of Channel 1 is associated with an increased contribution of Channel 2. Error bars represent standard deviation from *n* ≥ 3 independent experiments.

We interpret the ion‐dependent behavior using a two‐channel adhesive framework (Figure 5a). Channel 1 corresponds to polymer‐mica interactions, governed by electrostatic attraction between q‐BBP and the negatively charged mica surface, and modulated by charge screening and cation‐controlled surface accessibility. Channel 2 represents interpolymer cohesion mediated by multivalent anions, which can sustain adhesion even when Channel 1 is weakened.

**FIGURE 5:**
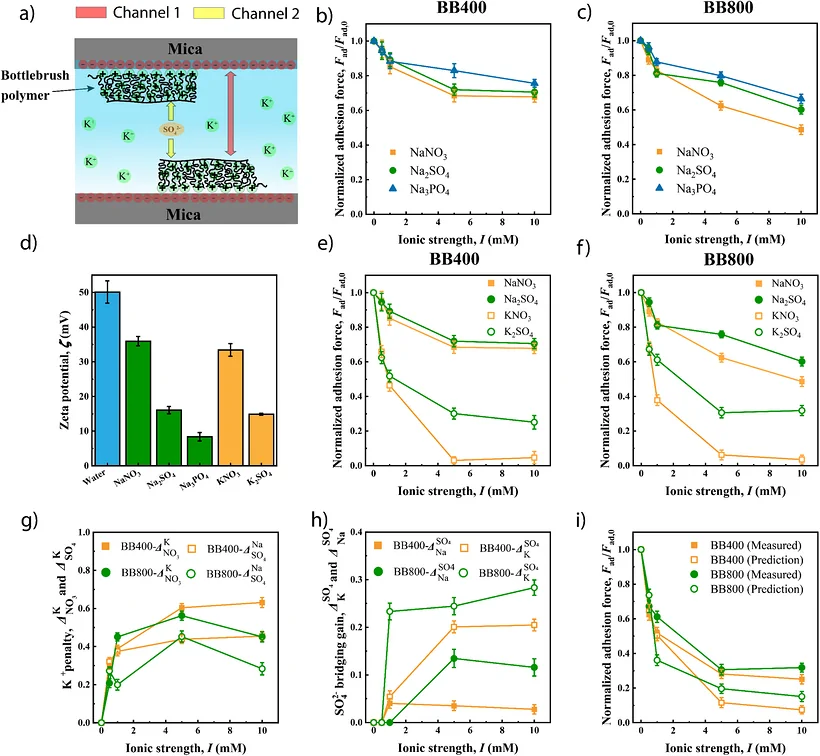
Ion‐specific control of adhesion in the symmetric q‐BBP/q‐BBP configuration.

We first examined the effect of anion identity using sodium salts (Figure 5b,c). Overall, adhesion decreases with increasing ionic strength for both polymers tested. In the low‐ionic‐strength regime (0–1 mm), NaNO_3_, Na_2_SO_4_ and Na_3_PO_4_ produce nearly identical reductions in adhesion for both BB400 and BB800. Phosphate hydrolysis at the measured pH of approximately 11 alters its speciation and lowers the effective ionic strength relative to the nominal value calculated by assuming ${\mathrm{PO}}_{4}^{3-}$. Nevertheless, the net ion‐specific contributions remain experimentally indistinguishable in this dilute regime. The initial adhesion reduction is therefore attributed primarily to electrostatic screening of Channel 1, which weakens polymer–mica attraction and reduces the ability of q‐BBP chains to access and bind exposed mica sites. Meanwhile, Channel 2 remains unresolved because the concentrations of multivalent anions are likely insufficient to produce a detectable population of cooperative interpolymer bridges.

As the ionic strength increases beyond 1 mm, the behavior becomes anion‐dependent. Adhesion retention increases systematically with anion valency (Na_3_PO_4_ > Na_2_SO_4_ > NaNO_3_), indicating the emergence of specific ion‐polymer interactions. ζ‐potential measurements (Figure 5d) show that multivalent anions reduce the apparent polymer charge more effectively than monovalent anions at equivalent ionic strengths. This observation is consistent with the stronger charge‐screening and/or charge‐neutralization effects arising from enhanced counterion association and ion–ion correlations [46, 47]. These effects would attenuate the direct electrostatic attraction between the cationic polymer and negatively charged mica (Channel 1). Consequently, the retention of adhesion in the presence of multivalent anions is consistent with an increased contribution from Channel 2, in which anion‐mediated interpolymer bridging partially compensates for the attenuation of direct polymer–mica interactions. On the other hand, this behavior can be further rationalized by differences in ion‐pairing structure governed by hydration thermodynamics. In the monovalent nitrate system, the weakly hydrated ${\mathrm{NO}}_{3}^{-}$ anion (Δ*G*_hyd_ ≈ −300 kJ mol^−1^) can approach the quaternary ammonium groups of q‐BBP more closely due to its lower dehydration penalty [26, 48]. This close association effectively neutralizes polymer charge and suppresses polymer‐mica interactions (Channel 1), but does not confer additional bridging interactions between chains, resulting in the lowest adhesion retention. In contrast, strongly hydrated multivalent anions including ${\mathrm{SO}}_{4}^{2-}$ and the phosphate species present under the experimental conditions, retain substantial hydration shells that influence their association with the quaternary ammonium groups [25, 26, 48, 49, 50]. This hydration‐mediated separation both screens the effective charge and introduces a steric and energetic barrier to direct polymer‐mica contact, further suppressing Channel 1. At the same time, their multivalent nature enables them to simultaneously interact with multiple polymer segments, effectively acting as molecular bridges that connect chains across the interface and activate interpolymer cohesion (Channel 2). This bridging compensates for the loss of direct polymer‐substrate binding and leads to enhanced adhesion retention.

The magnitude of this bridging response is further regulated by the polymer backbone length. BB800 exhibits a steeper baseline reduction in NaNO_3_ compared to BB400 (≈  50% vs ≈ 30%) consistent with its thicker, more diffuse interfacial layer (see Section 2.2) exposing more charged groups to ionic screening. At the same time, this extended conformation provides a higher density of accessible binding sites for multivalent anions, increasing the probability of cooperative interchain bridging. As a result, BB800 exhibits substantially greater adhesion recovery upon substitution of nitrate with multivalent anions, with increases of approximately 10%–15% in sulfate and 17%–18% phosphate compared with approximately 2%–4% and 4%–14%, respectively, for BB400 (Figure 5b,c).

While anions influence both interaction pathways, by screening polymer charge and modifying polymer‐mica interactions in Channel 1, and by enabling interpolymer bridging in Channel 2, cation identity primarily governs substrate accessibility in Channel 1. As seen in Figure 5e,f, replacing Na^+^ with K^+^ systematically reduces adhesion at a given anion. In the monovalent nitrate system (KNO_3_), adhesion drops to nearly zero between 5–10 mm. This near‐complete loss of adhesion is consistent with strong suppression of Channel 1 in the absence of a resolvable multivalent‐anion‐mediated contribution from Channel 2. In contrast, the K_2_SO_4_ system retains a finite adhesion plateau (≈ 30% of its zero salt value), demonstrating that sulfate‐mediated interpolymer bridging can partially compensate for the loss of polymer‐substrate interactions and preserve interfacial cohesion. Furthermore, K^+^ may facilitate partial displacement of polymer segments from the mica surface, producing a greater population of loops and tails extending into the solution. These more exposed conformations would increase the accessibility of cationic sites and favor sulfate‐mediated bridging between opposing polymer layers.

To quantitatively evaluate the K^+^ penalty and ${\mathrm{SO}}_{4}^{2-}$ bridging gain under different ionic backgrounds, we define pairwise contrasts at fixed ionic strength:

K^+^ penalty in a given anion background:

(1) $$\Delta_{NO_{3}}^{K}=A_{\mathrm{NaN}O_{3}}\;-A_{\mathrm{KN}O_{3}}$$

(2) $$\Delta_{SO_{4}}^{K}=A_{Na_{2}SO_{4}}\;-A_{K_{2}SO_{4}}$$

${\mathrm{SO}}_{4}^{2-}$ bridging gain in a given cation background:

(3) $$\Delta_{\mathrm{Na}}^{SO_{4}}=A_{Na_{2}SO_{4}}\;-A_{\mathrm{NaN}O_{3}}$$

(4) $$\Delta_{K}^{SO_{4}}=A_{K_{2}SO_{4}}\;-A_{\mathrm{KN}O_{3}}$$

where *A*_salt_ represents the normalized adhesion in the presence of various salt types. This analysis reveals that the K^+^ penalty is more pronounced in the ${\mathrm{NO}}_{3}^{-}$ background than in the ${\mathrm{SO}}_{4}^{2-}$ background ($\Delta_{NO_{3}}^{K}>\Delta_{SO_{4}}^{K}$ Figure 5g), and that the sulfate‐induced adhesion gain is significantly larger in the K^+^ background than in the Na^+^ background ($\Delta_{K}^{SO_{4}}>\Delta_{\mathrm{Na}}^{SO_{4}}$, Figure 5h). Together, these results indicate a strong coupling between substrate passivation and interpolymer bridging: suppression of polymer‐mica interactions enhances the relative contribution of bridging to interfacial cohesion, revealing a cooperative rather than additive interplay between the two pathways.

Backbone length further modulates these ion‐specific responses. Compared to BB400, BB800 exhibits both an earlier onset and a larger amplitude of the K^+^ penalty and ${\mathrm{SO}}_{4}^{2-}$ bridging. This behavior is consistent with its more extended interfacial conformation, which exposes a greater number of charged groups to ionic interactions and increases the likelihood of cooperative bridging.

To test whether cation and anion contributions are additive, we compared the experimental data with a model assuming independent effects:

(5) $$A_{K_{2}SO_{4}}^{\mathrm{pred}}=A_{\mathrm{KN}O_{3}}+\Delta_{\mathrm{Na}}^{SO_{4}}$$

where $A_{K_{2}SO_{4}}^{\mathrm{pred}}$ represents the predicted adhesion. This simple additive model systematically underpredicts the measured adhesion for both q‐BBPs (Figure 5i), demonstrating that sulfate‐mediated bridging is enhanced in the presence of K^+^, exceeding what is expected from independent contributions. The deviation from additivity emerges at lower concentrations for BB800, indicating that longer backbones amplify this cooperative effect. These results reveal a coupling between cation‐induced substrate passivation and anion‐mediated interpolymer bridging, in which suppression of substrate anchoring enhances the effectiveness of bridging interactions.

The divergence between Na^+^ and K^+^ arises from ion‐specific control of interfacial hydration and site accessibility at the mica surface, governed by coupled hydration thermodynamics and lattice matching. Despite its smaller bare radius, Na^+^ possesses a high charge density and a large hydration free energy (Δ*G*_hyd_ ≈ − 365 kJ mol^−1^) [48]. Because removing this hydration shell is energetically costly, Na^+^ retains a tightly bound hydration structure and predominantly forms outer‐sphere complexes with the mica surface [27, 28]. These hydrated ions remain separated from the lattice and effectively “float” above it, leaving the negatively charged ditrigonal cavities at the mica surface accessible for competitive adsorption of q‐BBP side chains (Scheme 2a). As a result, polymer–mica interactions (Channel 1) remain largely preserved.

**SCHEME 2:**
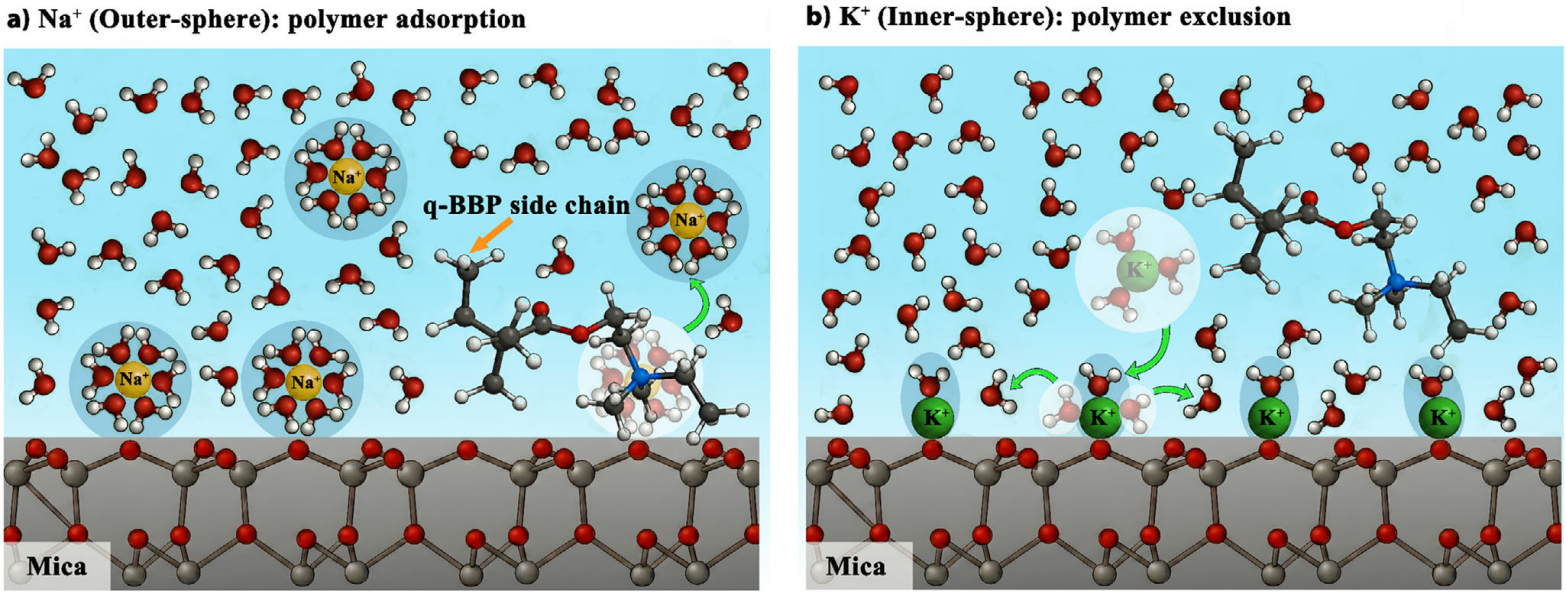
Proposed thermodynamic gating of adhesion via hydration‐controlled lattice accessibility. a, Na^+^ (outer‐sphere association): The strongly hydrated Na^+^ ion (yellow) retains a tightly bound hydration shell (blue shading) and remains separated from the mica surface, forming a labile outer‐sphere association. As a result, Na^+^ does not access the ditrigonal cavities, leaving negatively charged lattice sites available for the competitive adsorption of cationic q‐BBP side chains. b, K^+^ (inner‐sphere association): The weakly hydrated K^+^ ion (green) can partially shed its hydration shell and access the ditrigonal cavities, forming a more direct surface‐associated (inner‐sphere‐like) configuration. Occupation of these sites effectively blocks polymer access through a combination of steric exclusion and local electrostatic screening. As a result, polymer adsorption to the lattice is suppressed, and chains are displaced away from the surface, favoring extended conformations that can participate in interfacial bridging.

In contrast, K^+^ acts as an interfacial structure breaker. Its lower hydration penalty (Δ*G*_hyd_ ≈ −295 kJ mol^−1^) and favorable geometric match to the ditrigonal cavity enable partial dehydration and direct coordination with the lattice [28, 45, 48, 51]. By occupying these cavities, K^+^ effectively blocks access to adsorption sites and introduces both steric and electrostatic barriers that exclude q‐BBP side chains, thereby suppressing Channel 1 (Scheme 2b and Figure S2) [28, 33, 52, 53].

This hydration‐controlled passivation is supported by control experiments. In KNO_3_ solutions, bare mica‐mica interactions exhibit a pronounced short‐range repulsive hydration force that is absent in NaNO_3_ (Figure S3a), accompanied by a much faster decay of adhesion (Figure S3b). ζ‐potential measurements show negligible differences in electrophoretic mobility of q‐BBP in K^+^ and Na^+^ backgrounds (Figure 5d), indicating that the adhesion loss observed in K^+^ does not arise from the polymer charge difference but from surface passivation via lattice occupation.

A similar accessibility trend has been reported in chemically distinct interactions such as cation‐π binding [29]. Our results suggest that hydration‐controlled gating provides a unifying explanation: weakly hydrated cations (e.g., K^+^, ${\mathrm{NH}}_{4}^{+}$) more readily access and occupy interfacial binding sites, thereby attenuating interaction strength, whereas more strongly hydrated cations (e.g., Li^+^, Na^+^) remain shielded by persistent hydration shells and preserve the interaction strength. Treating the hydration layer as a steric and energetic barrier thus links mineral surface passivation and cation–π binding within a common thermodynamic framework.

Beyond surface passivation, cation identity further regulates interpolymer bridging (Channel 2) by controlling the thermodynamic availability of multivalent anions through bulk ion pairing. Ion pairing reflects a balance between the enthalpic cost of dehydration and the entropic gain from releasing structured water molecules [25, 26, 50]. Because both Na^+^ and ${\mathrm{SO}}_{4}^{2-}$ are strongly hydrated, high‐charge‐density ions, their association is entropically favored and relatively stronger, leading to a reduction in the population of free sulfate ions. In contrast, K^+^ interacts more weakly with sulfate and maintains a larger fraction of dissociated ${\mathrm{SO}}_{4}^{2-}$, consistent with the thermodynamic data showing higher association constants for Na^+^‐${\mathrm{SO}}_{4}^{2-}$ (log *K_a_* ≈ 0.95–1.11) than for K^+^‐${\mathrm{SO}}_{4}^{2-}$ (log *K_a_* ≈ 0.85–0.88) [54]. Molecular dynamics simulations further show that this strong association promotes the formation of Na^+^‐${\mathrm{SO}}_{4}^{2-}$ multiparticle clusters in solution [55, 56].

Although ion pairing remains weak under our experimental conditions (≤ 10 mm ionic strength), the same hierarchy persists. Using these constants as a guide, the estimated paired sulfate fraction at 10 mm is approximately 6%–8% in Na^+^ backgrounds versus 4%–5% in K^+^ backgrounds. This indicates that Na^+^ competes more effectively with q‐BBP for sulfate association, thereby reducing the availability of free ${\mathrm{SO}}_{4}^{2-}$ for interpolymer bridging.

Taken together, K^+^ exerts dual control over the adhesive landscape: it passivates the mica surface and suppresses the polymer‐substrate interactions (Channel 1), while simultaneously preserving a larger reservoir of bridging anions that sustain interpolymer cohesion (Channel 2). The combined effect leads to adhesion that exceeds predictions based on independent ion contributions, revealing a nonlinear coupling between interfacial hydration and bulk ion‐association equilibria.

## 3 Conclusion

This work shows that dissolved ions act as active regulators of interfacial mechanics rather than passive screening agents. Using bottlebrush polymer layers on mica as a force‐resolved model system, we identify a hydration‐governed mechanism that separates adhesion into two coupled pathways: polymer‐substrate anchoring and interpolymer cohesion. Within this framework, cation hydration controls access to surface binding sites, acting as a thermodynamic gate for polymer anchoring, while multivalent anions enable cohesion through interpolymer bridging across the confined interface.

The interplay between these pathways produces behaviors that cannot be explained by ionic strength alone. In particular, we observe non‐additive coupling between hydration‐controlled surface passivation and anion‐mediated bridging, giving rise to distinct adhesion regimes depending on electrolyte identity. These results establish that interfacial adhesion can be quantitatively tuned through ion‐specific hydration and association.

By explicitly resolving how cations and anions cooperatively regulate interfacial interactions, this work provides a physically grounded framework for electrolyte‐controlled adhesion in aqueous systems. More broadly, it highlights ion identity as a practical design parameter for modulating soft interfaces under confinement.

## 4 Experimental Section

### 4.1 Materials

The monomers 2‐(dimethylamino)ethyl methacrylate (DMAEMA) and 2‐(2‐bromoisobutyryloxy)ethyl methacrylate (BIEM) were purchased from Sigma‐Aldrich and passed through a basic alumina column prior to polymerization to remove inhibitors. Electrolytes (NaNO_3_, Na_2_SO_4_ Na_3_PO_4_, KNO_3_, and K_2_SO_4_; ≥ 99.99%, Sigma‐Aldrich Canada) were used as received. High‐grade ruby muscovite mica (Grade I, ASTM V‐1, S&J Trading Inc.) was used as the substrate for all surface force measurements. All aqueous solutions were prepared using ultrapure water (18.2 MΩ cm, TOC < 5 ppb; Arium Pro, Sartorius Canada) and filtered through a 0.22 µm PVDF membrane prior to use.

### 4.2 Atomic Force Microscopy (AFM) Imaging

Samples for AFM imaging were prepared on freshly cleaved ruby muscovite mica (Grade I, ASTM V‐1) to match the substrate conditions used in SFA measurements. A droplet of q‐BBP solution (50 µg mL^−1^) was deposited onto the mica and allowed to adsorb for 1 h. The substrate was then thoroughly rinsed with ultrapure water to remove non‐adsorbed polymer and dried at ambient temperature. Surface morphology was characterized by using an atomic force microscope (Dimension Icon/FastScan, Bruker) operating in PeakForce Tapping mode in air. Silicon tips mounted on silicon nitride cantilevers (nominal resonance 50–90 kHz; spring constant ≈ 0.4 N m^−1^) were used. For each condition, 2–4 independently prepared substrates were examined, with at least three regions imaged per substrate. Representative images are shown.

### 4.3 Surface Forces Apparatus (SFA) Measurements

Back‐silvered ruby muscovite mica sheets (thickness 1–3 µm) were glued onto cylindrical silica disks (*R* ≈ 2 cm) using an optical‐grade epoxy resin (EPON 1004F). The disks were mounted in the SFA chamber in a crossed‐cylinder geometry. The reference zero separation (*D* = 0) was established by bringing the bare mica surfaces into adhesive contact in air prior to each experiment. During measurements, a water reservoir was maintained in the chamber to ensure a saturated vapor environment and minimize evaporation.

### 4.4 Polymer Adsorption Protocols

Symmetric configuration (q‐BBP/q‐BBP): Polymer solution was injected between the mica surfaces and allowed to adsorb for 60 min. The surfaces were then rinsed 4–5 times with ultrapure water to remove unbound polymer, followed by reintroduction of ≈ 50 µL of pure water for measurements.

Asymmetric configuration (q‐BBP/mica): Adsorption was performed on a single mica surface prior to assembly. The lower surface was incubated in q‐BBP solution for 60 min and rinsed as described above, while the opposing surface remained polymer‐free. The two surfaces were then brought into contact.

Surface separation distance (*D*) was measured in real time using multiple‐beam interferometry (MBI) based on fringes of equal chromatic order (FECO). Interference fringes were digitized and analyzed using a custom MATLAB routine based on a three‐layer optical model. Normal interaction forces (*F*(*D*)/R) were determined from the deflection of a calibrated double‐cantilever spring and recorded during approach and separation at a constant speed of 1 nm s^−1^. Each condition was measured at multiple contact positions across at least three independently prepared surfaces.

### 4.5 Zeta Potential (ζ‐potential) Measurements

Electrophoretic mobility and ζ‐potential were measured using a Malvern Zetasizer Nano ZSP (Malvern Instruments Ltd., Worcestershire, UK). q‐BBP solutions (*C_p_* =  100 µg mL^−1^) were prepared in the specified electrolyte solutions to ensure sufficient scattering intensity. Measurements were conducted at 25°C using disposable folded capillary cells (DTS1060). ζ‐potential values were calculated from electrophoretic mobility using the Smoluchowski approximation. Reported values represent the mean ± standard deviation of at least three independent measurements.

## Author Contributions

**Chang‐Sheng Wang**: conceptualization, methodology, investigation, data curation, writing – original draft, writing – review and editing. **Hui Guo**: methodology, investigation, validation. **Hu Zhang**: investigation, methodology, conceptualization, writing – original draft, writing – review and editing, formal analysis. **Duy Anh Pham**: investigation, validation. **Nahid Hassanpour**: investigation, validation. **Wojciech Raj**: investigation, validation. **Qiang Peng**: investigation, validation. **Justine Saber**: investigation, validation. **Tzu‐Hsuan Huang**: investigation, validation. **Florina G. Halmac**: investigation, validation. **Xavier Banquy**: conceptualization, methodology, data curation, supervision, project administration, funding acquisition, writing – review and editing, writing – original draft.

## Conflicts of Interest

The authors declare no conflicts of interest.

## Supporting information




**Supporting File**: advs78071‐sup‐0001‐SuppMat.docx.

## Data Availability

The data that support the findings of this study are available on request from the corresponding author. The data are not publicly available due to privacy or ethical restrictions.
