# Supplementary material for: Nonlinear Ion‐Specific Control of Underwater Adhesion by Bottlebrush Polymer Interfaces
Source: Adv Sci (Weinh). 2026 Sep 28:e78071. Online ahead of print. doi: 10.1002/advs.78071 (PMC13620263; doi:10.1002/advs.78071)
Supplement: Supplementary file 1 [file ADVS-9999-e78071-s001.docx]

Supporting Information

**Nonlinear Ion-Specific Control of Underwater Adhesion by Bottlebrush Polymer Interfaces**

Chang-Sheng Wang, ^a‡^ Hui Guo, ^a‡^ Hu Zhang, ^a^ Duy Anh Pham,^b^ Nahid Hassanpour, ^a^ Wojciech Raj, ^a^ Qiang Peng, ^a^ Justine Saber, ^a^ Tzu-Hsuan Huang,^c^ Florina G. Halmac,^a^ Xavier Banquy ^abc*^

*^a^ Faculty of Pharmacy, Université de Montréal, Montréal, Québec H3T 1J4, Canada*

*^b^ Biomedical Engineering Institute, Faculty of medicine, Université de Montréal,* *Montréal, Québec, H3C 3J7, Canada*

*^c^ Department of Chemistry, Faculty of Art and Science, Université de Montréal, Montréal, Québec, H3C 3J7, Canada*

Corresponding author: Xavier Banquy
E-mail: xavier.banquy@umontreal.ca

**Table S1.** Synthesis conditions and structural parameters of the P(MMA-co-BiBEM) backbones. Summary of the ATRP polymerization conditions, including the monomer-to-initiator feed ratios, conversion rates, and the calculated degree of polymerization for the methyl methacrylate (*m*) and HEMA-TMS (*n*) repeating units 1.

|  | Molar ratio of (m+n)/Br on the initiator | Conversion  Ratio [%] | m^a)^ | n^b)^ | Total backbone units |
| --- | --- | --- | --- | --- | --- |
| P(MMA-co-HEMA-TMS)_418_ | 1000 | 19.5 | 110 | 99 | 418 |
| P(MMA-co-HEMA-TMS)_880_ | 2000 | 21.5 | 231 | 209 | 880 |

Note: ^a)^m: repeating unit number of MMA; ^b)^n: repeating unit number of HEMA-TMS.

**Table S2.** Molecular characteristics of macroinitiators and bottlebrush polymers. Number-average molecular weight (*M*_n_) and dispersity (PDI) of the ATRP-synthesized macroinitiators and the corresponding quaternized bottlebrush polymers (BB400 and BB800), as determined by gel permeation chromatography (GPC).

|  | ***M*_n_ (g/mol)** | **PDI** |
| --- | --- | --- |
| Macroinitiator-BB400 | 77000 | 1.07 |
| Bottlebrush-BB400 | 659220 | 1.28 |
| Macroinitiator-BB800 | 159000 | 1.22 |
| Bottlebrush-BB800 | 713440 | 1.34 |

**Scheme S1.** Repeated compression-separation cycles may promote the rearrangement of extended tail segments into more surface-associated, train-like conformations, leading to progressive layer compaction. This transition may occur in both asymmetric and symmetric configurations but is expected to be more pronounced in the symmetric configuration, consistent with its stronger cycle-dependent SFA response.

Scheme S2 Proposed effects of anion valency and countercation identity on the two adhesion channels of interfacial q-BBPs. In the K^+^ background, direct polymer-mica attraction (Channel 1) is strongly attenuated, whereas multivalent sulfate and phosphate species promote anion-mediated interpolymer bridging (Channel 2); monovalent nitrate produces no bridging. In the Na^+^ background, Channel 1 is comparatively retained, while sulfate and phosphate enhance Channel 2.

**Figure S1.** Asymmetric (q-BBP/mica) interaction forces in pure water. Representative SFA normal force–distance profiles, $F(D)/R$, measured between a bare mica surface and a q-BBP coated surface for **a,** BB400 and **b,** BB800. Profiles were collected at increasing polymer incubation concentrations ($C_{p}=25, 50, 100$ and $200 {\mu g mL}^{-1})$. Filled symbols denote the approach phase; open symbols denote separation (speed ${\text{1 }\text{nm s}}^{-1}$). The attractive onset distance $D_{\text{onset}}^{\text{att}}$ increases with $C_{p}$ and backbone length, consistent with the extension of partially adsorbed chains (bridging adsorption). Data represents mean trends from $n\geq3$ independent coatings per condition.

**Figure S2. Mechanical stability of the asymmetric (q-BBP/mica) interface under cyclic compression.** Evolution of the hard-wall thickness ($D_{\mathrm{hw}}$) versus compression cycle number for **a,** BB400 and **b,** BB800 in the asymmetric configuration. In contrast to the symmetric system, $D_{\mathrm{hw}}$ remains effectively invariant across consecutive cycles, independent of polymer concentration. This lack of hysteresis confirms that the polymer chains form a robust, irreversibly anchored layer on the mica substrate (stable Channel 1) that does not undergo significant conformational restructuring or desorption under compressive load.

**Figure S3.** Specific cation effects on bare mica-mica adhesion. **a)** Normal force-distance profiles between mica surfaces measured in $\mathrm{NaNO}_{3}$ and $\mathrm{KNO}_{3}$ solutions at an ionic strength of 10 mM. The $\mathrm{KNO}_{3}$ profile exhibits a distinct short-range repulsive slope (hydration cap) prior to contact, absent in $\mathrm{NaNO}_{3}$. **b)** Normalized adhesion force as a function of ionic strength. The adhesive minimum is consistently weaker and suppressed more rapidly $\mathrm{KNO}_{3}$ than in $\mathrm{NaNO}_{3}$. This confirms that the $K^{+}$ penalty arises from direct lattice passivation (inner-sphere complexation) even in the absence of polymer.
